# Successful Treatment of Primary Poor Graft Function After Haploidentical Hematopoietic Stem Cell Transplantation With Low‐Dose Decitabine Followed by Donor Lymphocyte Infusion and Eltrombopag

**DOI:** 10.1002/cam4.71636

**Published:** 2026-03-15

**Authors:** Jiaojiao Yuan, Junjie Cao, Peipei Ye, Tiantian Wang, Dong Chen, Shuangyue Li, Renzhi Pei, Juntao Zhang, Ying Lu

**Affiliations:** ^1^ The Affiliated People's Hospital of Ningbo University Zhejiang China; ^2^ Institute of Hematology, Ningbo University Zhejiang China

## Abstract

**Objective:**

Primary poor graft function (PGF) is a common and serious complication after haploidentical hematopoietic stem cell transplantation (haplo‐HSCT). This study retrospectively evaluates the efficacy and safety of a novel combination therapy consisting of low‐dose decitabine, donor lymphocyte infusion (DLI), and eltrombopag for the treatment of primary PGF subsequent to haplo‐HSCT.

**Methods:**

In this analysis of ten patients, decitabine was administered at a dose of 7 mg/m^2^/day for three consecutive days, followed by DLI on the fifth day and eltrombopag, which was started at 50 mg/day and titrated up to 150 mg. The primary endpoints of the study encompassed hematologic recovery.

**Results:**

Nine patients (90%) achieved a complete response with normalized blood counts, while one patient (10%) showed a partial response with transfusion independence. Adverse events included manageable graft‐versus‐host disease (GVHD) in four patients.

**Conclusion:**

These findings indicate that this triple therapy represents a promising approach for the management of primary PGF following haplo‐HSCT.

PGF followed by haplo‐HSCT is a clinical syndrome marked by sustained cytopenias despite the presence of complete donor chimerism. This condition significantly elevates the risk of infections, hemorrhage, and overall mortality. The incidence of PGF has been reported to range from 5% to 27% [1, 2, 3, 4]. Primary PGF arises from defects in stem cells, the bone marrow microenvironment, or post‐transplant immune suppression. Despite various therapies targeting these mechanisms, primary PGF often remains refractory, underscoring the critical need for novel therapeutic strategies for this high‐risk population.

This study retrospectively evaluated the efficacy and safety of a combination therapy using low‐dose decitabine, DLI, and eltrombopag for primary PGF after haplo‐HSCT. The cohort included 10 patients treated at the Affiliated People's Hospital of Ningbo University from January 2019 to December 2022, all of whom had undergone conditioning with fludarabine, antithymocyte globulin, and post‐transplant cyclophosphamide. The treatment protocol comprised the administration of low‐dose decitabine at 7 mg/m^2^ intravenously over three consecutive days (days 1–3). On the fifth day, DLI and eltrombopag therapy were commenced. Following DLI, all patients continued their prior cyclosporine‐based GVHD prophylaxis regimen, with the addition of a short‐course of methotrexate starting from day 1 after DLI. The methotrexate was administered at a dose of 10 mg/m^2^ once weekly for 4 consecutive doses. Eltrombopag was initially prescribed at a dose of 50 mg daily, with incremental increases of 25 mg every two weeks in the absence of improvement, up to a maximum daily dose of 150 mg. Upon achieving a substantial hematological response, the eltrombopag dosage was gradually tapered by 50% and subsequently discontinued.

Primary PGF was defined as hemocytopenia > 2 weeks, after day +28 in the presence of donor chimerism > 95%. Complete response (CR) was defined as almost normalization of peripheral blood counts (hemoglobin value > 100 g/L; platelets value > 50 × 10^9^/L; neutrophil count > 1.5 × 10^9^/L) for ≥ 7 consecutive days and partial response (PR) was defined as achievement of transfusion or granulocyte colony‐stimulating factor stimulation (G‐CSF) independence (hemoglobin value > 80 g/L; platelets value > 20 × 10^9^/L; neutrophil count > 0.5 × 10^9^/L). The study was conducted in accordance with the principles of the Declaration of Helsinki and was approved by the independent ethics committee of the Affiliated People's Hospital of Ningbo University. Written informed consent was provided by all patients.

The patients' clinical characteristics are shown in Table 1. The median age was 48.5 years (range 24–58). Before treatment, thrombocytopenia was present in 8 patients (80%), anemia in 6 (60%) and neutropenia in 5 patients (50%). Treatment was initiated a median of 64.5 days (range 12–201) after diagnosis of primary PGF. To mitigate the GVHD associated with DLI, we reduced the number of stem cells infused. The median counts of mononuclear cells, CD34+ cells, and CD3+ cells in the DLI were 3.21 × 10^8^/kg (range, 1.19–6.61), 0.44 × 10^6^/kg (range, 0.13–0.82), and 4.91 × 10^7^/kg (range, 2.32–8.94), respectively. Full blood counts (FBC) on the day of therapy may not accurately reflect the hematopoietic potency of the corresponding bone marrow due to repetitive and occasionally frequent transfusions or G‐CSF injection. As a result, the mean values of all FBC collected from each patient within the two weeks before treatment were used to compute baseline data. Nine patients had CR (90%) and one patient achieved PR (10%) after receiving therapy with this triple therapy. Baseline FBC values were 0.46 × 10^9^/L neutrophils (range 0.16–0.65), 60 g/L hemoglobin (range 56–66), and 10.5 × 10^9^/L platelets (range 4.2–14.0). Significant increases in neutrophils, platelets, and hemoglobin levels after treatment are shown in Figure 1. Neutrophils (Figure 1a) increased in all five patients with baseline neutropenia to a median of 3.11 × 10^9^/L (range 1.52–4.29, *p* = 0.0048, 95% CI 1.24–3.62) over a median of 18 days (range 10–20) after treatment. Seven patients (87.5%) with baseline thrombocytopenia showed a median increase in platelets (Figure 1b) over a median of 15 days (range 8–32) following intervention, reaching a median maximum value of 69 × 10^9^/L (range 36–87, *p* < 0.0001, 95% CI 43–70). A median rise in hemoglobin (Figure 1c) to median highest values of 109 g/L (range 90–135, *p* = 0.0008, 95% CI 33–70) was noted during a median of 20.5 days (range 9–84) in all six patients with baseline anemia after therapy. No serious hepatic or renal dysfunction or thrombosis was observed. Two patients developed grade I acute GVHD (aGVHD), one patient experienced grade III aGVHD and one patient developed mild chronic GVHD (cGVHD). By follow‐up, 8 patients had survived and 2 had died, with patient #6 dying of relapse and patient #10 discontinuing treatment for personal reasons. The median time after haplo‐HSCT for the whole cohort was 30.5 months (range 5.3–66.3). Among the eight surviving patients, all maintained their hematologic responses at the last follow‐up.

**TABLE 1:** Clinical characteristics.

Number	Gender (M/F)	Age (years)	Disease type and status	Donor Type	HLA match	ABO match(Donor/receptor)	Regimen (MAC/RIC)	Graft type	Cytopenias type	DLI			Response	Time to Response (days)			Acute GVHD	Chronic GVHD	Time from haplo‐HSCT (months)	State
										Mononuclear cells (10^8^ kg^−1^)	CD34+ cells (10^6^ kg^−1^)	CD3+ cells (10^**7**^ kg^**−**1^)		NE	HB	PLT				
1	M	53	AML(CR)	haploidentical	5/10	A+/A+	MAC	BM + PBSC	neutropenia/thrombocytopenia/anemia	3.77	0.46	4.98	CR	20	20	25	None	None	71.3	alive
2	M	33	ALL(CR)	haploidentical	5/10	B+/AB+	MAC	BM + PBSC	thrombocytopenia	6.61	0.13	8.94	CR	/	/	15	None	None	55.5	alive
3	F	39	AML(CR)	haploidentical	5/10	A+/O+	MAC	PBSC	neutropenia/thrombocytopenia/anemia	1.31	0.41	2.32	CR	10	9	12	Grade 1	None	36.6	alive
4	F	24	AML(CR)	haploidentical	5/10	A+/O+	MAC	PBSC	anemia	4.04	0.82	6.11	CR	/	84	/	Grade 1	None	43.0	alive
5	F	36	AML(CR)	haploidentical	5/10	O+/O+	MAC	PBSC	thrombocytopenia	3.25	0.48	4.84	CR	/	/	15	None	None	33.4	alive
6	F	56	AML(CR)	haploidentical	5/10	O+/A+	RIC	PBSC	neutropenia/thrombocytopenia	2.92	0.65	3.22	CR	10	/	17	None	None	7.9	dead
7	F	55	PMF	haploidentical	5/10	A+/O+	RIC	PBSC	neutropenia/thrombocytopenia/anemia	2.16	0.53	3.78	PR	19	13	32	None	Mild	34.4	alive
8	F	46	AML(CR)	haploidentical	5/10	O+/A+	MAC	BM + PBSC	neutropenia/thrombocytopenia	3.98	0.27	6.27	CR	17	/	8	None	None	57.0	alive
9	M	58	AML(CR)	haploidentical	6/10	A+/O+	MAC	PBSC	anemia	3.17	0.31	5.14	CR	/	27	/	None	None	31.4	alive
10	M	51	T‐LBL(CR)	haploidentical	5/10	O+/A+	MAC	PBSC	thrombocytopenia/anemia	1.19	0.38	2.56	CR	/	21	11	Grade 3	None	5.3	dead

Abbreviations: M, male; F, female; AML, acute myeloid leukemia; ALL, acute lymphoblastic leukemia; PMF, primary myelofibrosis; T‐LBL, T‐lymphoblastic cell lymphoma; MAC, myeloablative conditioning; RIC, reduced intensity conditioning; BM, bone marrow; PBSC, peripheral blood stem cells; CR, complete response; PR, partial response.

**FIGURE 1:**
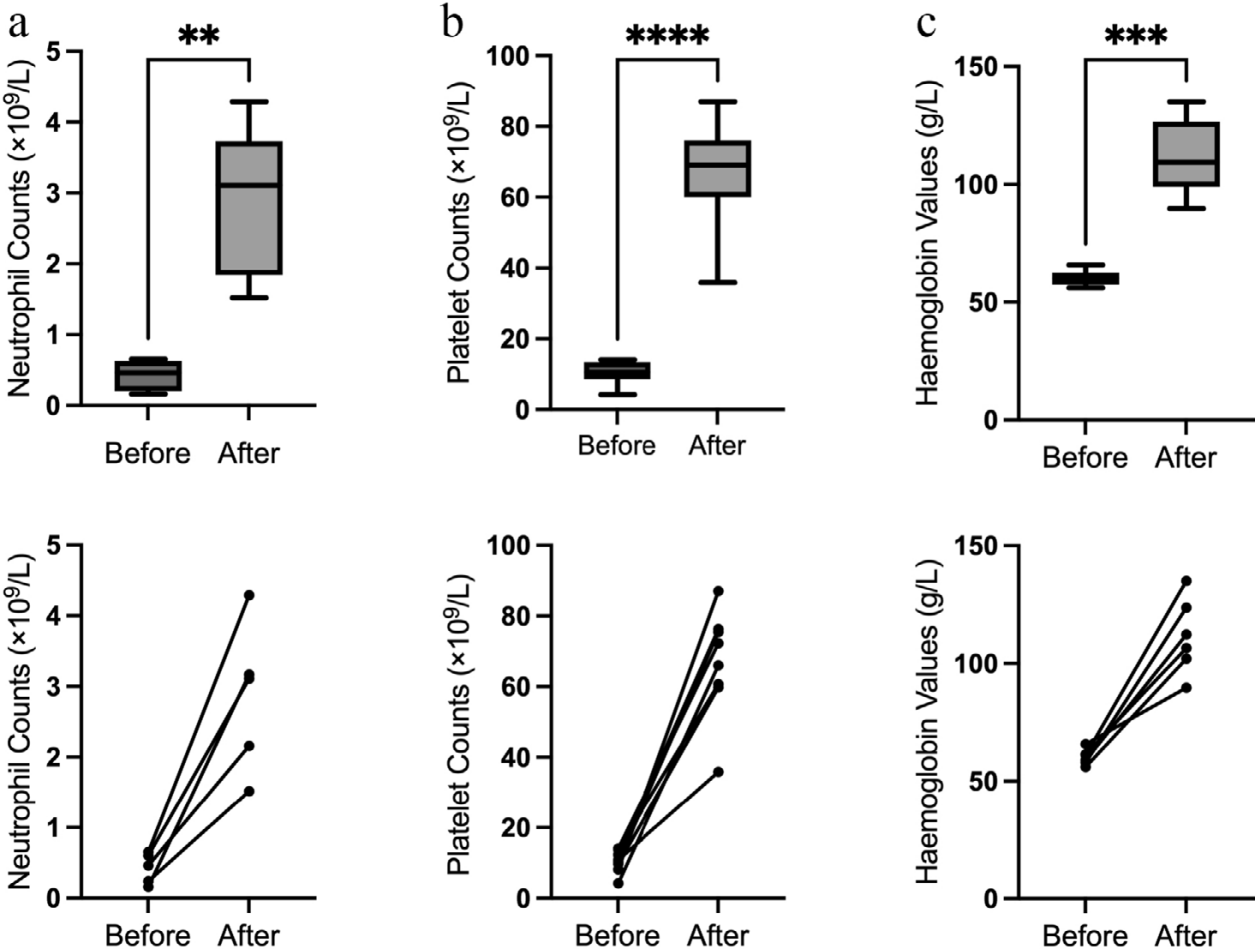
Figure 1: Hematologic recovery following combination therapy with low‐dose decitabine, donor lymphocyte infusion (DLI), and eltrombopag in patients with primary poor graft function (PGF) after haploidentical hematopoietic stem cell transplantation. (a) Neutrophil counts, (b) platelet counts, and (c) hemoglobin levels before and after treatment. Box plots show the median (central line), interquartile range (box), and range (whiskers). Statistical significance was determined using paired t‐tests: **p < 0.01, ***p < 0.001, ****p < 0.0001.

In the management of primary PGF, diverse therapeutic strategies exhibit distinct advantages and limitations. Monotherapy with either low‐dose decitabine or eltrombopag demonstrates limited efficacy for PGF and is primarily suitable for patients with isolated thrombocytopenia. The CD34^+^ selected stem cell boost effectively addresses the fundamental issue of stem cell insufficiency and minimizes the risk of GVHD by depleting mature T cells. However, its clinical application is limited by the requirement for donor remobilization and the complex, costly nature of immunomagnetic selection procedures, thereby reducing its feasibility for widespread implementation. Conversely, unmanipulated DLI offers a more accessible and cost‐effective alternative. It primarily facilitates hematopoietic recovery through immunomodulation and cytokine‐mediated enhancement of the bone marrow microenvironment, albeit with a considerable risk of GVHD. To address this challenge, our center has explored the implementation of reduced‐dose DLI in combination with adjunctive therapies to improve treatment efficacy. Within our cohort, four patients experienced GVHD, including one case of grade III aGVHD. Fortunately, all instances were successfully managed with standard immunosuppressive therapy. The observed high and durable CR rate, together with manageable GVHD, collectively suggest that the therapeutic benefits of reduced‐dose DLI outweigh its associated risks. Future research should focus on further optimizing DLI dosing and timing strategies to enhance therapeutic efficacy while minimizing the risk of GVHD.

The integrated regimen of low‐dose decitabine, DLI, and eltrombopag utilized in our study potentially capitalizes on synergistic mechanisms of action. Low‐dose decitabine has been demonstrated to enhance the bone marrow microenvironment and promote platelet recovery [5, 6, 7]. DLI contributes to this process through immune reconstitution and the secretion of hematopoietic growth factors [8, 9]. Eltrombopag, a thrombopoietin receptor agonist, directly stimulates megakaryocyte proliferation and differentiation via the JAK2/STAT5 signaling pathway [10, 11]. This multi‐targeted mechanism offers a more comprehensive therapeutic solution compared to alternative strategies, such as CD34^+^‐selected stem cell boosts or monotherapy with either agent alone.

Nevertheless, this study has several limitations. Its retrospective design without a control group may introduce bias and limit generalizability. The proposed mechanisms remain speculative due to the absence of biological correlative analyses, and the heterogeneous follow‐up duration (5.3–66.3 months) hampers assessment of long‐term durability. Future work should include mechanistic studies, longer and more uniform follow‐up, and multicenter randomized controlled trials to validate our findings.

In conclusion, the findings of our study indicate that the combined use of low‐dose decitabine, DLI, and eltrombopag represents a promising new therapeutic option for patients experiencing primary PGF following haplo‐HSCT.

## Author Contributions

J.Y. collected data and drafted the initial manuscript. J.C. and P.Y. reviewed the initial manuscript. T.W., D.C., S.L., and R.P. collected and provided patient clinical data. J.Z. supervised the work. Y.L. and J.C. assigned the protocol and critically revised the manuscript for relevant intellectual content. All authors contributed to the article and approved the submitted version.

## Funding

Ningbo Clinical Research Center for Ophthalmology (2022L003/2023‐Y8); Ningbo Medical Science and Technology Project (2022Y37); Zhejiang Province Medical and Health Science and Technology Project (2024KY1603); Yinzhou District Agricultural and Social Development Science and Technology Project (2022AS021); Ningbo University Teaching Research Project 2025 (JYXM2025025).

## Ethics Statement

The studies involving human participants were reviewed and approved by The Ethics Committee of the Affiliated People's Hospital of Ningbo University. Written informed consent to participate in this study was provided by the participants' legal guardian/next of kin.

## Conflicts of Interest

The authors declare no conflicts of interest.

## Data Availability

The data that support the findings of this study are available from the corresponding author upon reasonable request.
